# A systematic review of contaminants in donor human milk

**DOI:** 10.1111/mcn.13627

**Published:** 2024-01-24

**Authors:** Sionika Thayagabalu, Nicole Cacho, Sandra Sullivan, John Smulian, Adetola Louis‐Jacques, Marie Bourgeois, Henian Chen, Wasana Weerasuriya, Dominick J. Lemas

**Affiliations:** ^1^ Department of Health Outcomes and Biomedical Informatics, College of Medicine University of Florida Gainesville Florida USA; ^2^ Department of Pediatrics, Division of Neonatology University of California Davis California USA; ^3^ Envision Healthcare, HCA Florida North Florida Hospital Gainesville Florida USA; ^4^ Department of Obstetrics and Gynecology, College of Medicine University of Florida Gainesville Florida USA; ^5^ Center for Perinatal Outcomes Research, College of Medicine University of Florida Gainesville Florida USA; ^6^ Department of Public Health University of South Florida Tampa Florida USA

**Keywords:** breast milk, contaminants, donor milk

## Abstract

Donor human milk (DHM) from a milk bank is the recommended feeding method for preterm infants when the mother's own milk (MOM) is not available. Despite this recommendation, information on the possible contamination of donor human milk and its impact on infant health outcomes is poorly characterised. The aim of this systematic review is to assess contaminants present in DHM samples that preterm and critically ill infants consume. The data sources used include PubMed, EMBASE, CINAHL and Web of Science. A search of the data sources targeting DHM and its potential contaminants yielded 426 publications. Two reviewers (S. T. and D. L.) conducted title/abstract screening through Covidence software, and predetermined inclusion/exclusion criteria yielded 26 manuscripts. Contaminant types (bacterial, chemical, fungal, viral) and study details (e.g., type of bacteria identified, study setting) were extracted from each included study during full‐text review. Primary contaminants in donor human milk included bacterial species and environmental pollutants. We found that bacterial contaminants were identified in 100% of the papers in which bacterial contamination was sought (16 papers) and 61.5% of the full data set (26 papers), with the most frequently identified genera being *Staphylococcus* (e.g., *Staphylococcus aureus* and coagulase‐negative *Staphylococcus*) and *Bacillus* (e.g., *Bacillus cereus*). Chemical pollutants were discovered in 100% of the papers in which chemical contamination was sought (eight papers) and 30.8% of the full data set (26 papers). The most frequently identified chemical pollutants included perfluoroalkyl substances (six papers), toxic metal (one paper) and caffeine (one paper). Viral and fungal contamination were identified in one paper each. Our results highlight the importance of establishing standardisation in assessing DHM contamination and future studies are needed to clarify the impact of DHM contaminants on health outcomes.

## Key messages

- Research regarding chemical contamination in donor human milk (DHM) is limited and needs to be studied further to draw appropriate conclusions on reducing potential risks for infants.
- Parents/guardians should be educated on the availability of DHM as a supplemental feeding option and made aware of the current research in the field.
- DHM use requires further standardisation both within the United States and abroad. This standardisation should ensure that risks are not overemphasised and DHM is a cost‐effective, accessible resource as a short‐term intervention when used appropriately as part of optimal lactation and breastfeeding support.

## Graphical abstract

This systematic review urges continued research in the field of contaminants and donor human milk (DHM) to delineate the risks and benefits of its use in premature and critically ill infants.  Our review reveals traces of chemicals, including PFAs and caffeine, within DHM. Bacteria, viruses and fungi are generally removed from DHM via pasteurisation.

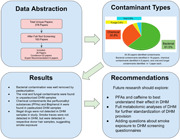

## 1 INTRODUCTION

Preterm infants receive nutrition via formula milk, mother's own milk (MOM) and/or donor human milk (DHM). The World Health Organization (WHO) and the American Academy of Pediatrics (AAP) recommend pasteurised DHM as the preferred feeding method for preterm infants when the availability of MOM is limited (Perrin et al., 2020). Consumption of DHM has been associated with outcomes including reduced incidence of necrotising enterocolitis (NEC) in preterm infants (Kantorowska et al., 2016), reduced NICU stay (Yu et al., 2019) and improved rates of breastfeeding at discharge, which can be expected to lead to positive health impacts in recipient infants (Shenker et al., 2023). DHM use also reduces the consumption of formula milk, a well‐known risk factor for NEC in preterm infants (Picaud, 2022). Although a larger number of studies have reported on contaminants in infant formula, including Cronobacter species, lead and cadmium (Mielech et al., 2021; NACMCF, 2022), information on DHM contamination and the potential impact on infant health outcomes is poorly characterised.

Provision of DHM for preterm infants occurs through a series of regulatory practices for screening, collection, processing and distribution to protect against contamination (Shenker et al., 2021). Screening for DHM is completed through self‐reported lifestyle questionnaires and blood tests that identify blood‐borne infections (e.g., HIV, syphilis, hepatitis B, hepatitis C) (Spatz, 2018). Potential mothers are excluded if the use of illegal drugs or tobacco products is detected in milk samples (Abrams et al., 2017). Collection of DHM occurs most commonly through milk banks like the Human Milk Banking Association of North America (HMBANA), and donors are given detailed instructions regarding storage and shipment of milk (Abrams et al., 2017). Processing of DHM is completed via holder pasteurisation, high‐temperature‐short‐time pasteurisation, high‐pressure processing, ultraviolet‐C irradiation or vat pasteurisation (Kim et al., 2023; Moro et al., 2019). Distribution occurs from milk banks to NICUs within hospitals, which also receive storage guidelines (Abrams et al., 2017). Although the WHO is currently developing global DHM guidelines to reduce harmful contamination (WHO, 2022), DHM provision is not standardised across different entities (Speer, 2022).

Inconsistencies in DHM guidelines exist within the United States and internationally. For example, Pennsylvania mandates screening and pasteurisation by state law, Maryland treats DHM as tissue banks and Texas has requirements set by the state health department. Moreover, DHM banks are not licensed or regulated under state law in many states; most existing state‐level policies in the United States focus on DHM insurance coverage rather than processing and handling (Rose et al., 2022; Speer, 2022). Global guidance on DHM standardisation and use is currently limited as well, though the WHO is notably working to strengthen global guidelines (WHO, 2022). For example, Vietnam requires milk banks to operate under established national hospital quality standards. Brazil has created a national DHM banking system using glass and mayonnaise jars for DHM collection and manual pasteurisation rather than automated pasteurisation for DHM processing. Kenya has developed its own operating guidelines based on hazard analysis and quality assurance of DHM (Tyebally Fang et al., 2021). Though such methods are operational, worldwide standardisation of DHM collection is useful  to ensure that DHM is equally safe for consumption by infant populations. Global DHM standardisation efforts are currently in the works by entities like the European Milk Bank Association (Weaver et al., 2019), HMBANA (Spatz, 2018) and Prolacta (Thibeau & Ginsberg, 2018).

In this analysis, we completed a systematic review of studies focused on identifying contaminants in DHM for preterm infants. We also aim to compare contaminants present in the DHM both prepasteurization and postpasteurization. Further, we add to the existing literature with a push for stronger DHM regulation and urge further standardisation to assist the ongoing WHO efforts.

## 2 METHODS

### 2.1 Inclusion and exclusion criteria

Briefly, we included experimental studies that identified contaminants within DHM. Our systematic review followed the guidance of the Preferred Reporting Items for Systematic Reviews and Meta‐Analyses (PRISMA). We excluded studies that: (a) did not have the abstract and/or full paper written in English; (b) were review articles, case studies or editorials; (c) were repeats that made it through the initial Covidence screening software; (d) focused on milk from other vertebrates and not human donors; (e) focused on maternal or infant outcomes and (f) investigated maternal perspectives towards DHM. Inclusion criteria included studies that: (a) had the abstract and/or full paper written in English; (b) were novel experimental studies and (c) reported contaminants identified in DHM.

### 2.2 Search strategy

Electronic searches were conducted in PubMed, Embase, CINAHL and WoS databases to identify publications with a focus on DHM and contamination appearing between January 1, 2010, and July 31, 2021. A librarian with expertise in databases aided in the search process. Additional literature was extracted from the references of publications retrieved via our initial search terms and through expert suggestions. The papers were analysed and tagged for data extraction using Covidence. The goal of title and abstract screening was to selectively identify papers that related to donor milk metabolomics in humans; inclusion and exclusion criteria were used to reduce the paper count to 163. Disagreements regarding eligibility were discussed to reach a consensus among the reviewers. Papers were also added based on clinical expert recommendations. Table S1 summarises the search strategy for the paper‐extraction process.

### 2.3 Data extraction

Data extraction was conducted from June 2021 to December 2022 using the Covidence software. The 26 included papers were reviewed by a team of two reviewers (S. T. and D. L.). Author names, publication dates and paper titles were extracted. During the evaluation process, the papers were further tagged with contaminant type, factor(s) utilised for comparison between samples in a study, presence of pasteurised milk samples and location of the study. Contaminant types included bacteria, viruses, fungi and chemicals. Each DHM contaminant identified was categorised through clinical expert consultation and recommendations. Our team utilised open discussion to ensure that descriptions were precise, and tags were understandable to any future audience interested in our paper.

## 3 RESULTS

**Figure 1:**
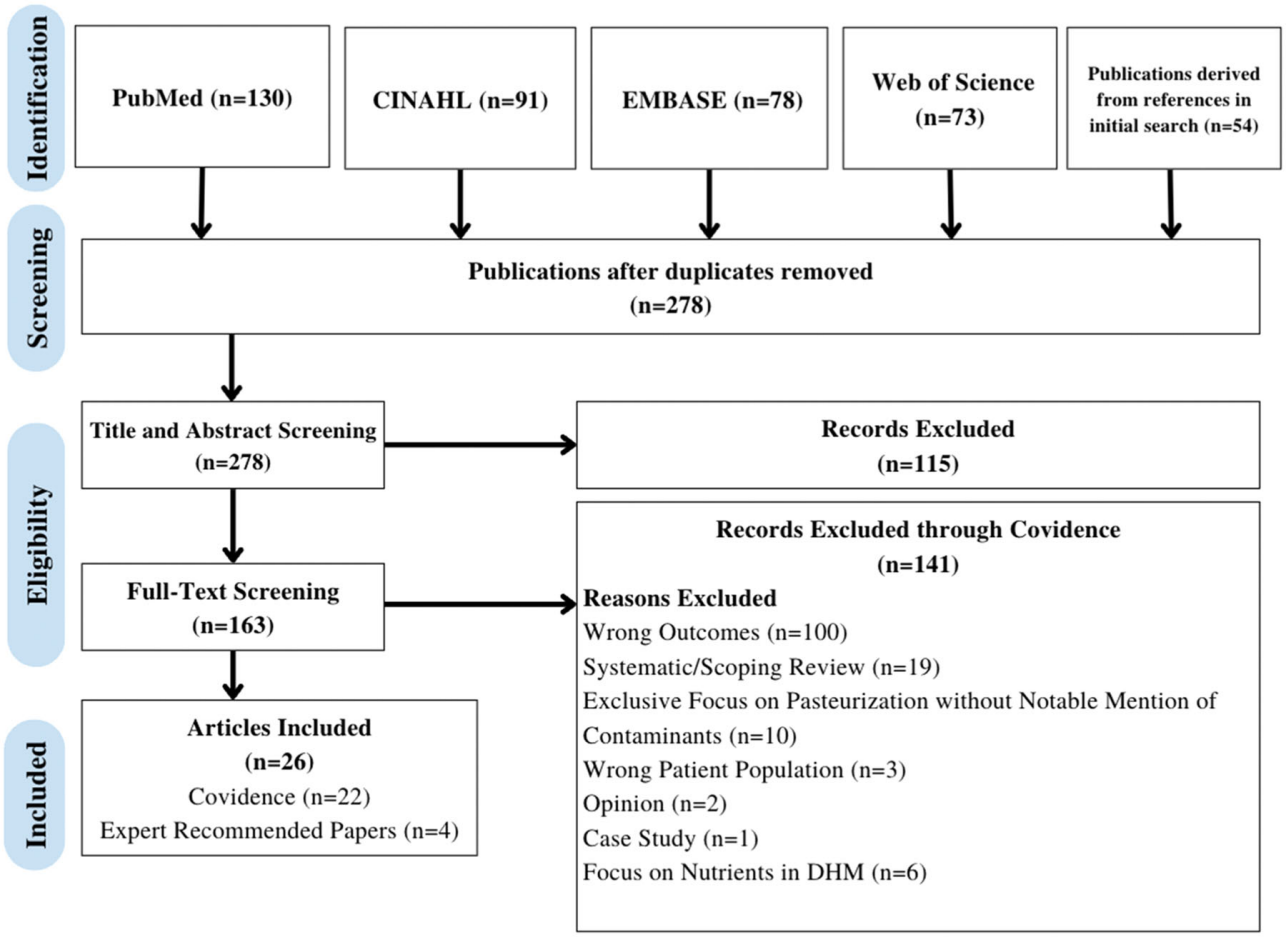
PRISMA diagram. A literature search of PubMed, EMBASE, CINAHL and Web of Science (WoS) resulted in 372 papers. Fifty‐four papers were extracted from the references of the publications retrieved through our initial search. There were 130 papers from PubMed/MEDLINE, 91 papers from CINAHL, 78 papers from Embase and 73 papers from WoS. Two hundred and seventy‐eight papers were screened for eligibility after duplicates were removed through Covidence. One hundred and fifteen studies were initially excluded during title and abstract screening for irrelevance. One hundred and sixty‐three studies were assessed through a full‐text review and 141 were removed for wrong outcomes (100), systematic/scoping review (19), extensive focus on pasteurisation (10), wrong patient population (3), opinion (2), case study (1) and focus on nutrients in DHM (6). Four papers were added via expert recommendation to yield 26 papers in our data set. The graphic above characterises the search and selection process.

The workflow is shown in Figure 1. Briefly, we completed a literature search of PubMed, EMBASE, CINAHL and Web of Science (WoS), resulting in 372 papers. There were 54 papers extracted from the references of the publications retrieved through our initial search. There were 130 papers from PubMed/MEDLINE, 91 papers from CINAHL, 78 papers from Embase and 73 papers from Web of Science. After duplicates were removed through Covidence, 278 papers were screened for eligibility. A total of 115 studies were initially excluded during title and abstract screening for irrelevance. The remaining 163 studies were assessed through a full‐text review and 141 were removed for the following reasons: wrong outcomes (100), systematic/scoping review (19), exclusive focus on pasteurisation without notable mention of contaminants (10), wrong patient population (3), opinion (2), case study (1) and focus on benefits and nutrients in DHM (6). Wrong outcomes were characterised as outcomes focusing on infant health or infant responses to DHM. The remaining 22 studies were included in this systematic review, including three papers obtained from the data set of 54 papers from existing paper references. Through clinical expert recommendation, our team added four papers, resulting in a final data set of 26 papers in total.

Studies that reached the final review stage came from a vast array of nations. The studies were conducted in the United States (4), Italy (4), Australia (4), Canada (1), Spain (2), Brazil (1), India (1), France (1), China (1), Norway (1), Greece (1), Netherlands (1), Israel (1) and Ireland (1). Two studies incorporated samples from different nations. One of these two studies assessed donor human milk in Spain and Brazil (Oliveira et al., 2020). The other assessed the quality of donor human milk in 52 nations (van den Berg et al., 2017). Papers assessing the effects of pasteurisation on donor human milk composition were commonly identified. With regard to pasteurisation, 17 papers definitively indicated that postpasteurisation testing was conducted as a part of the milk analysis, as specified in Table 1. Table 2 further describes the eight studies which sought to identify the effects of DHM pasteurisation on contamination. The general trend was that bacterial contamination decreased with pasteurisation (Landers & Updegrove, 2010; Mandru et al., 2019; Singh et al., 2017; de Waard et al., 2018) unless the bacteria were spiked into or naturally contaminated the sample postpasteurisation (Almutawif et al., 2019; Mallardi et al., 2022).

**Table 1:** Distribution of contaminants in DHM publications. This table characterises the specific contaminants identified in each of the 26 studies included in this systematic review. It includes details on the nation in which each study was conducted, characterises the specific comparators utilised in each study and indicates the presence of pasteurisation methods for clinical purposes.

Citation	Source	Paper title	Contaminant type	Contaminant subtype	Country	Comparison factor(s) in the study	Presence of pasteurised samples
Aceti et al. (2021)	Covidence	Exposure to perfluoroalkyl substances through human milk in preterm infants	Chemical	Perfluoroalkyl substances	Italy	Birth status (preterm or full‐term)	Yes
Almutawif et al. (2017)	Covidence	A retrospective audit of bacterial culture results of donated human milk in Perth, Western Australia	Bacterial	Coagulase negative *Staphylococcus*, Acinetobacter, *Staphylococcus aureus*	Australia	Time at which donation was made (2007–2011)	Yes
Almutawif et al. (2019)	Covidence	*Staphylococcus aureus* enterotoxin production in raw, holder‐pasteurised and ultraviolet‐C‐treated donated human milk	Bacterial	*Staphylococcus aureus*, enterotoxins	Australia	Treatment (raw, holder‐pasteurised and ultraviolet‐C‐treated)	Yes
Almutawif et al. (2019)	Covidence	*Staphylococcus aureus* enterotoxin production in raw and pasteurised milk: The effect of selected different storage durations and temperatures	Bacterial	*Staphylococcus aureus*, enterotoxins	Australia	Storage (durations and temperatures)	Yes
Clifford et al. (2021)	Covidence	What are optimal bacteriological screening test cut‐offs for pasteurised donor human milk intended for feeding preterm infants?	Bacterial	Enterobacterales, *Bacillus cereus*	Australia	Compliance to established milk discard guidelines	Yes
Demers‐Mathieu et al. (2020)	Covidence	Impact of vaccination during pregnancy and staphylococci concentration on the presence of Bacillus cereus in raw human milk	Bacterial	*Bacillus cereus*, *Staphacoccus aureus*, coagulase‐negative staphylococci	Nevada, USA	Vaccination	No
Elmekkawi et al. (2018)	Covidence	Impact of neonatal intensive care unit admission on bacterial colonisation of donated human milk	Bacterial	Coagulase negative staphylococci, *Micrococcus* spp., *Corynebacterium* spp., *Propionibacterium* spp., nonpathogenic *Neisseria* spp., *Bacillus* spp. (other than *B. cereus* and *B. anthracis*), yeast, *Staphylococcus aureus*; β‐haemolytic streptococcus groups A, B, C and G, *Haemophilus* spp., *Streptococcus* spp., *Bacillus cereus*, *Bacillus anthraci*s, *Enterococcus* spp., Enterobacteriaceae, afermenting Gram‐negative bacilli	Canada	NICU admission and storage duration	No
Escuder‐Vieco et al. (2016)	Covidence	Breast milk and hair testing to detect illegal drugs, nicotine and caffeine in donors to a human milk bank	Chemical	Nicotine, cotinine, caffiene	Spain	Breast milk and hair samples	Not specified
Hutchings et al. (2021)	Covidence	Antimicrobial effect of Zn^2+^ ions governs the microbial quality of donor human milk	Bacterial	*Staphylococcus epidermidis*, coagulase‐negative Staphylococci, *Bacillus cereus*	Israel	Antibacterial treatment with zinc	Yes
Lindemann (2004)	Covidence	Characteristics of breast milk and serology of women donating breast milk to a milk bank	Bacterial	*Staphylococcus aureus*, klebsialla species, enterobacter species, serratia species, *E. coli*	Norway	Pasteurisation	Yes
Mallardi et al. (2022)	Covidence	New operating approach to limit bacillus cereus contamination of donor human milk	Bacterial	*Bacillus cereus*	Italy	Improved hygiene and pasteurisation	Yes
Mandru et al. (2019)	Covidence	Bacterial content of fortified and unfortified holder pasteurised donor human milk during prolonged refrigerated storage	Bacterial	Not specified	United States	Pasteurisation and storage duration	Yes
Masson et al. (2019)	Covidence	Bacteriological screening of breast milk samples destined to direct milk donation: Prospective evaluation between 2007 and 2016	Bacterial	*Staphylococcus aureus*, methicillin‐resistant *S. aureus*, *Klebsiella pneumoniae*, *Klebsiella oxytoca*, *Escherichia coli*, *Enterobacter cloacae*, *E. coli* producing extended‐spectrum β‐lactamases	France	Compliance to existing bacterial standards in DHM	Not specified
Min et al. (2020)	Covidence	The nucleic acid positive rate and genotype distribution of human cytomegalovirus in human milk banks in China	Viral	Viral DNA including gB1, gB2 and gB3	China	Location of bank	No
Oliveira et al. (2020)	Covidence	Essential and toxic elements in human milk concentrate with human milk lyophilizate: A preclinical study	Chemical	Aluminium, arsenic, cadmium, chromium, iron, mercury, manganese, nickel, lead, selenium, tin and thallium	Brazil and Spain	Essential versus toxic metals	Yes
Papachristou et al. (2020)	Covidence	Microbiological control of donor breast milk—Criteria for acceptance or rejection	Bacterial	Conjugase negative staphylococcoci, *Enterococcus*, *Staphylococcus aureus*, Streptococcus, Gram‐negative bacteria (Klebsiella, proteus, serratia, *E. coli* and pseudomona)	Greece	Birthweight and preterm status of donor's baby	Yes
Serrano et al. (2021)	Covidence	Concentrations of perfluoroalkyl substances in donor breast milk in Southern Spain and their potential determinants	Chemical	PFAs, PFHpA, PFOA, PFNA, PFHxA, PPFTrDA, perfluorooctane sulphonate (PFOS)	Spain	PFA type	Yes
van den Berg et al. (2017)	Covidence	WHO/UNEP global surveys of PCDDs, PCDFs, PCBs and DDTs in human milk and benefit‐risk evaluation of breastfeeding	Chemical	Dibenzo‐*p*‐dioxins (PCDDs), polychlorinated dibenzofurans (PCDFs) and polychlorinated biphenyls (PCBs)	52 nations	Countries	Not specified
de Waard et al. (2018)	Covidence	Holder‐pasteurised human donor milk: How long can it be preserved?	Bacterial	*Bacillus cereus, B. mycoides, B. thuringiensis, Corynebacterium tuberculostearicum, Neisseria elongata, Rothia dentocariosa, Rothia mucilaginosa, S. capitis, S cohnii, S epidermidis, S. hominis, S. warneri, Streptococcus mitis, Streptococcus oralis, Streptococcus parasanguinis* and *Streptococcus sanguis*	Netherlands	Pasteurisation	Yes
Barbarossa et al. (2013)	Aceti et al. (2021)	Perfluoroalkyl substances in human milk: A first survey in Italy	Chemical	PFOS, PFOA	Italy	Number of births for woman	No
Landers and Updegrove (2010)	Demers‐Mathieu et al. (2020)	Bacteriological screening of donor human milk before and after holder pasteurisation	Bacterial	Coagulase‐negative *Staphylococcus*, GNRs, Enterococcus, α‐Streptococcus, *Bacillus* sp., *S. aureus*, Diphtheroids	Texas, USA	Pasteurisation	Yes
Serafini et al. (2003)	Hutchings et al. (2021)	Microbiological quality of human milk from a Brazilian milk bank	Bacterial	*Staphylococcus* spp., *Streptococcus* spp., yeasts and moulds and Enterobacteriaceae	Brazil	Thermal treatment	Yes
Hartle et al. (2018)	Expert recommendation	Chemical contaminants in raw and pasteurised human milk	Chemical	PBDE47, PCB153, ppDDE and MEHHP (phthalate metabolite), chlorpyrifos, BPA, permethrin	United States	Pasteurisation	Yes
Abdallah et al. (2020)	Expert recommendation	Concentrations of perfluoroalkyl substances in human milk from Ireland: Implications for adult and nursing infant exposure	Chemical	PFAs	Ireland	PFA type	Not specified
Micco et al. (1995)	Expert recommendation	Evaluation of ochratoxin A level in human milk in Italy	Fungal	Ochratoxin A	Italy	Ochratoxin presence	No
Singh et al. (2017)	Expert recommendation	Bacteriological analysis of donor human milk in milk bank in an Indian setting	Bacterial	Gram‐positive bacilli, coagulase‐negative staphylococci, Gram‐negative bacilli	India	Hospital or bank, pasteurisation	Yes

**Table 2:** Analysis of studies exploring prepasteurised and postpasteurised milk samples. This table details the change in bacterial or chemical contaminant types and amounts because of pasteurisation. Bacterial contamination was seen to decrease with pasteurisation unless the sample was spiked upon pasteurisation. Chemical contamination did not change significantly because of pasteurisation.

Citation	Paper title	Type of contaminant	Method of external contamination, if any	Impact on postpasteurization samples	Main takeaways
Almutawif et al. (2019)	*Staphylococcus aureus* enterotoxin production in raw, holder‐pasteurised and ultraviolet‐C‐treated donated human milk	Bacterial	Intentional spiking of milk	Increased bacterial count	Samples were spiked with *S. aureus*, which suggests an external source of bacterial contamination would be required to increase colony counts in the holder‐pasteurised milk. Raw milk is capable of suppressing *S. aureus* growth compared to pasteurised DHM.
Almutawif et al. (2019)	*Staphylococcus aureus* enterotoxin production in raw and pasteurised milk: The effect of selected different storage durations and temperatures	Bacterial	Intentional spiking of milk	Increased bacterial count	*S. aureus* growth increased in pasteurised samples when compared to raw samples upon spiking.
Mallardi et al. (2022)	New operating approach to limit *Bacillus cereus* contamination of donor human milk	Bacterial	N/A	Decreased bacterial count	Reduced amount of DHM postpasteurization samples discarded upon an improved hygiene approach.
Mandru et al. (2019)	Bacterial content of fortified and unfortified holder pasteurised donor human milk during prolonged refrigerated storage	Bacterial	N/A	Decreased bacterial count	Pasteurised donor human milk was tested for the effects of fortifier status and storage time on bacterial growth. They were found to not be significantly associated with increased bacterial growth.
de Waard et al. (2018)	Holder‐pasteurised human donor milk: How long can it be preserved?	Bacterial	N/A	Decreased bacterial count	Pasteurised donor human milk is safe for infant consumption regarding milk storage time. 9.8% of the samples in the study had bacterial growth when the follow‐up samples were cultured, but none showed sequential contamination. There were no significant decreases in macronutrients and energy content over 8 months.
Landers and Updegrove (2010)	Bacteriological screening of donor human milk before and after holder pasteurisation	Bacterial	N/A	Decreased bacterial count	Routine holder pasteurisation resulted in 93% of milk samples in this study showing no growth on routine bacterial cultures.
Singh et al. (2017)	Bacteriological analysis of donor human milk in milk bank in an Indian setting	Bacterial	N/A	Decreased bacterial count	Hospital donations were more contaminated in comparison to milk bank donations, both pre‐ and postpasteurization. The most common organisms isolated in prepasteurized samples were Gram‐positive bacilli, coagulase‐negative staphylococci and Gram‐negative bacilli. The most common organisms in postpasteurized samples were Gram‐positive bacilli and CONS. No Gram‐negative bacilli were isolated from postpasteurized samples.
Hartle et al. (2018)	Chemical contaminants in raw and pasteurised human milk	Chemical	N/A	Slightly decreased chemical count	19 of 23 tested chemicals appeared in the prepasteurized milk samples and 18 of 23 tested chemicals appeared in the postpasteurized milk samples of this study. Pasteurisation did not affect the presence of most of the chemicals.

**Figure 2:**
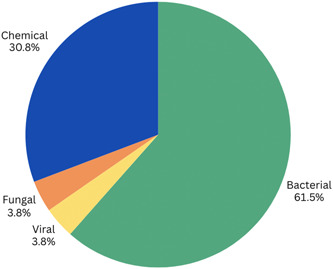
Contaminants found in donor human milk. *Note*: All 26 papers identified contaminants in donor milk. Bacterial contaminants were identified in 16 of the 26 papers. Chemical contaminants were identified in six papers. Viral contaminants were identified in one paper and fungal contaminants were also identified in one paper.

Figure 2 details the contaminant types identified within the paper set. It also highlights the most frequently identified contaminants in each of the four contaminant categories (bacterial, chemical, viral and fungal). Table 1 characterises the specific contaminants identified in each of the 26 studies and details the nation(s) in which data collection occurred for each study. Bacterial contaminants were sought for specifically in 16 of the 26 papers and identified in all 16 papers. Primary bacterial contaminants identified include *Staphylococcus aureus*, *Bacillus cereus* and coagulase‐negative *Staphylococcus* (CoNS). Of the 26 papers, nine reported traces of *S. aureus*, six reported traces of *B. cereus* and seven reported traces of CoNS. Other bacteria identified in the papers less frequently include Acinetobacter, Enterobacterales and Propionibacterium. Viral contaminants were found in less prominent amounts, being identified in one paper. Viral DNA from Human cytomegalovirus (CMV), including gB1, gB2 and gB3, was found within 46.4% of donated, unpasteurised milk samples in China in one included paper (Min et al., 2020). Fungal contaminants were also found in less prominent amounts, being identified in one paper. This study identified the fungal contaminant ochratoxin A in 22 of the 111 donated, unpasteurised milk samples collected (Micco et al., 1995).

Environmental chemical pollutants, including but not limited to perfluoroalkyl substances (PFAs), polychlorinated dibenzodioxins (PCDDs), polychlorinated dibenzofurans (PCDFs) and polychlorinated biphenyls (PCBs) were found in the six papers in which they were sought. Manganese and selenium are trace minerals that were found in low, acceptable concentrations. Lead was present in low concentrations in samples within the paper that discussed metal toxicity (Oliveira et al., 2020). One paper discussed traces of caffeine, illegal drugs and second‐hand smoke in DHM. No illegal drugs were found in the samples taken within the study and caffeine was identified in 50% of the DHM samples. Nicotine and cotinine traces were identified in the hair samples of 33.3% of the donors followed in this study, but not in respective donor milk samples (Escuder‐Vieco et al., 2016). Table 3 details the specific chemical contaminants that were sought and analysed within each of the 8 papers exploring chemical contamination. It provides additional details regarding the samples and concentrations in which certain identified chemicals were found.

**Table 3:** Analysis of studies exploring chemical contamination. This table details the specific chemical contaminants that were analysed and sought within each of the eight papers exploring chemical contamination. It provides additional details regarding the samples/concentrations in which certain identified chemicals were found.

Citation	Paper title	Chemicals tested for	Relevant details from the study
Aceti et al. (2021)	Exposure to perfluoroalkyl substances through human milk in preterm infants	PFAs	Amount of PFASs in 10 preterm and 10 DHM samples was evaluated and estimated daily intake (EDI) was calculated. Median EDI was 6.4–28.96 ng/kg/day.
Escuder‐Vieco et al. (2016)	Breast milk and hair testing to detect illegal drugs, nicotine and caffeine in donors to a human milk bank	Nicotine, caffeine, morphine, cocaine, cannabis, amphetamines, codeine, methadone and derived substances	36 donors were tested. Nicotine and cotinine were found in 33.3% of the hair samples and no mention was made of these substances in the DHM samples. However, the researchers warn that high levels of smoke exposure found in hair may be correlated with infant health effects from donor's milk. Caffeine was found in 50% of the DHM samples (18 of 36 donors).
Oliveira et al. (2020)	Essential and toxic elements in human milk concentrate with human milk lyophilizate: A preclinical study	Aluminium, arsenic, cadmium, chromium, iron, mercury, manganese, nickel, lead, selenium, tin and thallium	Tested by inductively coupled plasma‐mass spectrometry (ICP‐MS). Upon donated breast milk direct lyophilization, manganese (+0.80 μg/L) and selenium concentration (+6.74 μg/L) increased while lead concentration (−6.13 μg/L) decreased.
Serrano et al. (2021)	Concentrations of perfluoroalkyl substances in donor breast milk in Southern Spain and their potential determinants	PFAs	PFAs identified in 24%–100% of the DHM samples. PFHpA was detected in 100% of samples, followed by PFOA (84%), PFNA (71%), PFHxA (66%) and PFTrDA (62%). Perfluorooctane sulphonate (PFOS) was detected in 34% of donors. Media PFA concentration in donors was 87.67 ng/L.
van den Berg et al. (2017)	WHO/UNEP global surveys of PCDDs, PCDFs, PCBs and DDTs in human milk and benefit‐risk evaluation of breastfeeding	Polychlorinated dibenzo‐*p*‐dioxins (PCDDs), polychlorinated dibenzofurans (PCDFs) and polychlorinated biphenyls (PCBs) and the sum of dichlorodiphenyltrichloroethanes (ΣDDTs)	Levels of PCDDs and PCDFs were highest in India, parts of Europe and parts of Africa. PCB levels were highest in East and West Europe. High ΣDDTs were found in less industrialised countries.
Barbarossa et al. (2013)	Perfluoroalkyl substances in human milk: A first survey in Italy	PFAs including perfluorooctane sulphonate (PFOS) and perfluorooctanoic acid (PFOA)	Measured concentrations ranged between 15 and 288 ng/L for PFOS and between 24 and 241 ng/L for PFOA.
Hartle et al. (2018)	Chemical contaminants in raw and pasteurised human milk	23 chemicals including the persistent organic pollutants (POPs) polychlorinated biphenyls (PCBs), polybrominated diphenyl ethers (PBDEs), dichlorodiphenyltrichloroethane (DDT) and dichlorodiphenyldichloroethylene (DDE)	19 of 23 tested chemicals appeared in the prepasteurized milk samples and 18 of 23 tested chemicals appeared in the postpasteurized milk samples of this study. Pasteurisation did not affect the presence of most of the chemicals. Chlorpyrifos and BPA were found in all samples and permethrin was found in 90% of the samples. Chlorpyrifos and permethrin were degraded significantly by pasteurisation.
Abdallah et al. (2020)	Concentrations of perfluoroalkyl substances in human milk from Ireland: Implications for adult and nursing infant exposure	10 perfluoroalkyl substances (PFASs)	Four PFASs (PFOA, PFNA, PFHxS and PFOS) were detected in 16 DHM samples in Ireland. PFOA was found in the highest levels at a median of 0.10 ng/mL.

## 4 DISCUSSION

Pasteurised DHM is a rational infant feeding substitute for MOM and formula milk; however, information on potential contaminants in DHM remains poorly characterised. In this study, we completed a systematic review to assess contaminants present in DHM. We found that the primary contaminants in donor human milk included bacterial species and environmental pollutants. The most frequent bacteria were defined by the genera *Staphylococcus* (e.g., *S. aureus* and coagulase‐negative *Staphylococcus*) and *Bacillus* (e.g., *B. cereus*). Primary dietary contaminants identified include caffeine and heavy metal. Chemical contaminants were discovered in 30.8% of the papers overall and in 100% of the papers searching for chemical contaminants. Collectively, our results demonstrate a need for further standardisation of DHM and future study regarding the impact of such contaminants on infant health.

The diverse array of nations in which our included studies were conducted in is a notable point of discussion. As Table 1 demonstrates, the studies included in our data set were from various nations, indicating that there is a promising call for change in DHM protocol yet little standardisation worldwide. There were studies in North America (5), South America (2), Europe (11), Australia (4) and Asia (2). Although there are milk banks in Africa (Tyebally Fang et al., 2021), there were no studies included in our review that were conducted in Africa, suggesting limited DHM research studies in the region. Researchers should incorporate efforts to understand how DHM is utilised in regions within Africa to better address the topic globally as advancements by the WHO are underway (WHO, 2022). Only two studies incorporated samples from multiple nations. One of these studies assessed DHM in Spain and Brazil (Oliveira et al., 2020). The other assessed the quality of DHM in 52 nations (van den Berg et al., 2017), being the most inclusive study in our data set in terms of the number of nations that data collection covered. It is notable that regional and global DHM standardisation efforts are in the works by entities like the European Milk Bank Association (Weaver et al., 2019), HMBANA (Spatz, 2018) and Prolacta (Thibeau & Ginsberg, 2018). Our results demonstrate the need for continued international research efforts to inform and shape the advancements made by these entities to promote an understanding of these contaminants in DHM and to strengthen standardisation of DHM.

The main purpose of pasteurisation is to inactivate and reduce biological contaminants, including bacteria and viruses to the limits of detection. Our analysis found that bacterial contaminants were significantly reduced via pasteurisation and samples with notable postpasteurised bacterial contamination were discarded (Mallardi et al., 2022). Improved donor education and hygiene reduced contamination outbreaks upon pasteurisation and minimised disposal of postpasteurised samples (Mallardi et al., 2022), emphasising the importance of postpasteurisation testing at the NICU level as a method of ensuring safety to the highest extent. We also found that the two papers discussing DHM samples with detected viral DNA and fungal contamination, respectively, studied unpasteurised DHM. Specifically, Min et al. reported that viral DNA from human cytomegalovirus (CMV) was present in nearly 50% of the donated, unpasteurised milk samples, which is generally consistent with the CMV rates in the general population (Min et al., 2020). Pasteurisation inactivates virus activity and CMV would only be expected to be of concern when donor human milk is being used raw (Min et al., 2020). Regarding fungal contamination, the study which found ochratoxin A in 22 of the 111 donated, unpasteurised DHM samples reports that pasteurisation and handling improvements could reduce this risk (Micco et al., 1995). Overall, screening and processing of DHM decrease the risk of virus and fungi presence within DHM, indicating generally effective removal via pasteurisation.

Our study also identified chemical contaminants in DHM that included PFAs, PCDDs, PCDFs, bisphenol A (BPA), phthalates and polychlorinated biphenyls (PCBs). PCDDs, PCDFs and PCBs are often released by industrial and combustion processes like pesticide manufacturing, bleaching and metal processing; they enter the body via food consumption (Srogi, 2008). Existing data suggests that dioxins, BPA and phthalate exposure via human milk cause endocrine and metabolic disruptions (Lucaccioni et al., 2021; Pant et al., 2022; White & Birnbaum, 2009; Yan et al., 2009). Dioxins, a component of PCDDs, remain in fat stores and are linked with heart disease, cancer, diabetes, reproductive problems (early menopause and decreased testosterone) and reduced immunity (White & Birnbaum, 2009). BPA and phthalates are chemicals utilised in plastics that can enter the infant through various ways, including maternal exposure or plastic bottle feeding (Lucaccioni et al., 2021; Pant et al., 2022; Yan et al., 2009). As expected, pasteurisation does not significantly eliminate chemical contaminants (Hartle et al., 2018), presenting a potential risk when it comes to DHM consumption with heavy chemical contaminants. Future research is needed to quantify the impact of chemical contaminants within DHM on infant health outcomes.

Our team also evaluated the presence of illegal drugs, tobacco and alcohol in DHM. We did not find any illegal drug traces in DHM in the studies included in our analysis. However, we did include a study where second‐hand smoke, nicotine and cotinine traces were detected in hair samples, but not in the respective pasteurised DHM samples (Escuder‐Vieco et al., 2016). These results highlight the potential for noninvasive collection of biospecimens such as hair to supplement DHM screening to ensure collected DHM does not reflect exposure to tobacco products. Though our data set did not include any reports of alcohol traces in DHM, it is also important to study and standardise alcohol traces and thresholds in DHM collection and use worldwide. Donors are screened for illegal drugs, tobacco and alcohol via lifestyle questionnaries. These questionnaries have been identified as generally reliable for illicit drug use, though limitations arise from second‐hand smoke and caffeine consumption. To ensure that DHM collection does not include tobacco exposure, our results suggest it may be important to inquire about the smoking habits of partners during milk collection (Escuder‐Vieco et al., 2014). Collectively, our results demonstrate that screening for illegal drugs and tobacco has sufficiently limited the detection of these compounds in DHM.

An important observation from our analysis was the identification of caffeine in DHM. Caffeine is present in a variety of popular beverages, including tea, sports drinks, cocoa, chocolate, soda and coffee (Abalo, 2021). Coffee is one of the most consumed beverages worldwide (Abalo, 2021), illustrating the significance of its health effects in society and the importance of considering it for further study as a potential contaminant in DHM. Among 36 participants, Escuder‐Vieco et al. found that caffeine traces were identified in 50% of the DHM samples collected (2016). Interestingly, caffeine is a common medication for premature infants in NICU settings (Bauer et al., 2021). Excessive maternal caffeine intake may cause infant irritability and poor sleeping patterns, but no effect was noted with moderate intake of caffeinated beverages (Bauer et al., 2021). The AAP categorises caffeine as a maternal medication rather than a food, suggesting benefits in moderate consumption (Bauer et al., 2021). Thus, the effects of residual caffeine from DHM may not be detrimental but should be considered as a point of discussion with the family and medical team. Collectively, our results suggest that DHM screening questionaries should include questions focused on quantifying caffeinated beverages and food consumption as part of the lifestyle questionaries (Escuder‐Vieco et al., 2014).

DHM improves rates of MOM at discharge (Shenker et al., 2023) and reduces the consumption of formula milk, a well‐known risk factor for NEC in preterm infants (Picaud, 2022). The United States Food and Drug Administration states that systematic reviews of powdered infant formula report a relatively high contamination rate (2%–15%) of Cronobacter species (NACMCF, 2022). Moreover, lead and cadmium contamination have been identified in several infant formulas globally (Mielech et al., 2021). Our results reveal that lead concentrations in a study exploring toxic elements in DHM samples were low and acceptable (Oliveira et al., 2020). DHM samples with bacterial contamination are generally disposed of in NICU settings due to postpasteurization testing, which is something that is not conducted for formula milk, attributing to the high Cronobacter rates (NACMCF, 2022) and increased NEC (Picaud, 2022). Overall, it is of value to better understand contamination in DHM for characterisation as a potentially safer alternative for preterm infants.

Our study has both limitations and strengths. A limitation of our study is that our team only extracted studies that had abstracts in English, which may limit our global implications. Another limitation of our analysis is that there is heterogeneity in existing studies on DHM contamination. This is true across the nation(s) in which studies were conducted, the method of processing, use of pasteurisation and the analysis of samples. A standardised metabolite extraction process was not generally followed. For example, some papers screened specifically for a certain species of bacteria and did not examine viral or chemical contaminants. The best way to standardise this is to do complete metabolomic analyses of DHM. A strength of our study is that we were able to identify specific contaminants that should be further explored when creating global guidelines for DHM. We recommend future research regarding substances like PFAs and caffeine to best understand their effect on DHM. Standardisation in the field of contaminants and DHM will help to delineate the risks and benefits of its use in premature and critically ill infants.

Current DHM guidelines are not consistent with various governing entities. The United States focuses most policies on DHM insurance coverage rather than processing and handling (Rose et al., 2022; Speer, 2022). Global guidance on DHM standardisation and use is currently limited as well, though the WHO is notably working to strengthen global guidelines (WHO, 2022). Vietnam has established hospital quality standards, Brazil utilises manual pasteurisation methods and Kenya employs hazard analysis and quality assurance strategies for DHM provision (Tyebally Fang et al., 2021). Though DHM standardisation efforts are being made by entities like the European Milk Bank Association (Weaver et al., 2019), HMBANA (Spatz, 2018) and Prolacta (Thibeau & Ginsberg, 2018), further worldwide standardisation of DHM collection is necessary to ensure that all donor milk is equally safe for consumption by infant populations. With this health outcomes data, future legislation to promote stronger donor milk screening practices is attainable. Our team has initiated such research and passed a law in the Florida legislature to alleviate costs for donor human milk and make it accessible. With more standardisation comes most costs, and it is important to alleviate those stresses for patients and their families. More information is available at the following link (https://hscweb3.hsc.usf.edu/blog/2022/05/16/collaborative-effort-across-floridas-medical-schools-results-in-a-statute-expanding-medicaid-coverage-to-include-donor-breastmilk/).

## 5 CONCLUSION

Donor human milk requires a stronger evaluation of its components so researchers and clinicians may better understand the role that contaminants play in infant nutrition and health outcomes. Contaminants have been commonly recognised and identified in donor human milk, but there is no standardised way of assessing donor human milk quality and safety. Some future directions include conducting a systematic review to explore what literature exists about the effect of human donor milk on infant health, promoting the passage of legislation to mandate stronger donor milk screening practices, and conducting a metabolomic analysis of donor milk. Mothers should be educated on the availability of DHM as a supplemental feeding option and made aware of the current research in the field. Understanding donor milk is a multifaceted research effort that requires collaboration between the public, DHM banks and hospitals.

## AUTHOR CONTRIBUTIONS

Sionika Thayagabalu, Dr. Nicole Cacho and Dr. Dominick Lemas conceptualised and designed the study, drafted the initial manuscript and critically reviewed and revised the manuscript. All authors approved the final manuscript as submitted and agreed to be accountable for all aspects of the work.

## CONFLICT OF INTEREST STATEMENT

The authors declare no conflict of interest.

## Supporting information

Supporting information.

## Data Availability

Data sharing is not applicable to this article as no new data were created or analysed in this study.
