# Supplementary material for: A systematic review of contaminants in donor human milk
Source: Matern Child Nutr. 2024 Jan 24;20(2):e13627. doi: 10.1111/mcn.13627 (PMC10981490; doi:10.1111/mcn.13627)
Supplement: Supplementary file 1 — Supporting information. [file MCN-20-e13627-s001.docx]

Supplementary Tables

| Table 1. Search terms and databases | |
| --- | --- |
| Databases used for literature search | PubMed, Embase, Web of Science, and CINAHL |
| NICU/Preterm | "Intensive Care Units, Neonatal” OR "NICU" OR "newborn ICU" OR "neonatal ICU" OR "neonatal intensive care unit" OR "newborn intensive care unit" OR "infant, premature” OR "neonatal prematurity" OR "premature infants" OR "preterm infants" OR "premature birth" OR "premature birth" OR "premature labor" OR "neonatal prematurity" |
| AND | |
| Donor Milk | "donor milk" OR "donor human milk" OR "donor breastmilk" OR "donor breast milk" OR "donated breast milk" OR "donated breastmilk" OR "donated milk" OR "donated human milk" OR "human milk donation" OR "breast milk donation" OR "breastmilk donation" OR "milk donation" OR "pasteurized human milk" OR "pasteurized breastmilk" OR "pasteurized breast milk" |
| AND | |
| Contaminants | “contaminant” OR ”adulterant”  OR OR "prescription drug" OR "over the counter" OR "Nonprescription Drugs" OR "nonprescription drug" OR "Substance-Related Disorders” OR "substance abuse" OR "substance use" OR "substance usage" OR “alcohol” OR "marijuana" OR "cannabis" OR "metals, heavy" OR "heavy metal*" OR "lead" OR "arsenic" OR "mercury" OR "cadmium" OR "environmental exposure" OR "environmental monitoring" OR "hazardous substances" OR "hazardous substance*" OR "pesticides" OR “pesticide” OR "agrochemicals" OR "agrochemical*" OR "environmental pollution" OR ”pollutant” OR "occupational exposure" OR "occupational exposure" OR "environmental exposure" OR "environmental exposure" OR "bacteria” OR "bacterium" OR "bacteria*" OR "microbe" OR "communicable diseases" OR "infections" OR "infection*" OR "infectious" OR "communicable” |

**Supplementary Table 1**

**Search terms and databases.** PubMed, WOS, Embase, and CINAHL were used for literature search. Search keywords include words/phrases related to NICU/Preterm, donor milk, and contaminants. Boolean search was employed. Keywords within the same group related to “OR” logic while that between groups related to “AND” logic. Additionally, we restricted search fields to title and abstract, as well as subject headings for some keywords.
